# Identification of *Wolbachia*-Responsive miRNAs in the Small Brown Planthopper, *Laodelphax striatellus*

**DOI:** 10.3389/fphys.2019.00928

**Published:** 2019-07-24

**Authors:** Lei Liu, Kai-Jun Zhang, Xia Rong, Ya-Ying Li, Huai Liu

**Affiliations:** Key Laboratory of Entomology and Pest Control Engineering, College of Plant Protection, Southwest University, Chongqing, China

**Keywords:** *Laodelphax striatellus*, *Wolbachia*, microRNA, endosymbiont, insect-symbiont interaction

## Abstract

*Laodelphax striatellus* is naturally infected with the *Wolbachia* strain *w*Stri, which induces strong cytoplasmic incompatibility of its host. MicroRNAs (miRNAs) are a class of endogenous non-coding small RNAs that play a critical role in the regulation of gene expression at post-transcriptional level in various biological processes. Despite various studies reporting that *Wolbachia* affects the miRNA expression of their hosts, the molecular mechanism underlying interactions between *Wolbachia* and their host miRNAs has not been well understood. In order to better understand the impact of *Wolbachia* infection on its host, we investigated the differentially expressed miRNAs between *Wolbachia*-infected and *Wolbachia*-uninfected strains of *L. striatellus*. Compared with uninfected strains, *Wolbachia* infection resulted in up-regulation of 18 miRNAs and down-regulation of 6 miRNAs in male, while 25 miRNAs were up-regulated and 15 miRNAs were down-regulated in female. The target genes of these differentially expressed miRNAs involved in immune response regulation, reproduction, redox homeostasis and ecdysteroidogenesis were also annotated in both sexes. We further verified the expression of several significantly differentially expressed miRNAs and their predicted target genes by qRT-PCR method. The results suggested that *Wolbachia* appears to reduce the expression of genes related to fertility in males and increase the expression of genes related to fecundity in females. At the same time, *Wolbachia* may enhance the expression of immune-related genes in both sexes. All of the results in this study may be helpful in further exploration of the molecular mechanisms by which *Wolbachia* affects on its hosts.

## Introduction

*Wolbachia* is a maternally inherited endosymbiotic bacteria that infects with 40% of terrestrial arthropod species (Zug and Hammerstein, 2012). It draws attention by manipulating the reproduction of host in arthropod species. For example, cytoplasmic incompatibility (CI) is the best-known reproductive phenotype and result in early embryonic lethality when males infected by *Wolbachia* mating with uninfected females or females carrying different *Wolbachia* strains (Laven, 1957; Yen and Barr, 1971). In addition to reproductive regulation, a growing body of researches have shown that *Wolbachia* also affect many aspects of arthropod host, including the expression of immune genes in many arthropod hosts (Kambris et al., 2009; Thomas et al., 2011; Pan et al., 2012; Rances et al., 2012; Ye et al., 2013; Joshi et al., 2017). In fact, *Wolbachia* can affects the immune response of insects to a variety of pathogens, including infections against bacteria, viruses, and parasites (Kambris et al., 2009, 2010; Moreira et al., 2009; Bian et al., 2010; Mousson et al., 2010; Hughes et al., 2011; Wong et al., 2011; Pan et al., 2012; Eleftherianos et al., 2013; Gupta et al., 2017; Ant et al., 2018; Monsanto-Hearne and Johnson, 2018; Thomas et al., 2018).

MicroRNAs (miRNAs) exist in invertebrates and vertebrates, which play an important role in the regulation of gene expression at post-transcriptional level by combining with target mRNA complementarily (Lee et al., 1993; Reinhart et al., 2000; Lagos-Quintana et al., 2001). It takes part in lots of important physiological processes, including immune response (Lee and Hyun, 2014), responding to bacterial infection (Lourenco et al., 2013), reproduction (Zhang et al., 2016), and germline stem cell differentiation (Eun et al., 2013). In fact, some studies also showed that insect miRNAs are widely involved in host-microorganism interactions (Asgari, 2011; Wu et al., 2013; Slonchak et al., 2014; Li et al., 2015, 2018; Liu et al., 2015; Xing et al., 2016).

In recent years, *Wolbachia* regulating host genes expression through disturbing the expression of host miRNAs were reported (Hussain et al., 2011; Osei-Amo et al., 2012; Zhang et al., 2013, 2014; Mayoral et al., 2014; Rong et al., 2014). In *Aedes aegypti*, *Wolbachia* induce the expression of aae-miR-2940, which up-regulates the expression of arginine methyltransferase 3 and metalloprotease gene, as well as down-regulates the expression of DNA methyl-transferase gene, which was critical for its colonization and efficient maintenance of its density in host (Hussain et al., 2011; Zhang et al., 2013, 2014). In *Ae. aegypti* cells, Argonaute 1 distribution to the nucleus was blocked by *Wolbachia* via upregulating the expression of miR-981 (Hussain et al., 2013). Researchers also found *Wolbachia* induced the expression of aae-miR-12 to downregulate the expression of monocarboxylate transporter MCT1 and DNA replication licensing factor MCM6 genes that were critical for its persistence in *Ae. aegypti* cell line (Osei-Amo et al., 2012). In *Tetranychus urticae*, *Wolbachia* infection significantly suppresses expression of miRNAs, and the target genes of *Wolbachia*-responsive miRNAs involve in lysosome function and apoptosis in both sexes, in the meantime, it may regulate reproduction in females (Rong et al., 2014). All of these studies suggest that miRNAs may play a key role in *Wolbachia*-host interaction.

The small brown planthopper (SBPH), *Laodelphax striatellus*, is one of the most serious agricultural pests that feeds on the phloem sap of several important crops, such as rice, wheat and corn. It is also an insect vector, and transmits plant viruses by feeding on healthy and diseased plants (Zhang et al., 2007; Li et al., 2011). SBPH is naturally infected with the *Wolbachia* strain *w*Stri, which induces CI strongly (Noda et al., 2001). In this study, we inquired the effects of *Wolbachia* on the genes expression of *L. striatellus* by comparing miRNA expression levels in infected females (FI) and uninfected females (FUI), and infected males (MI) and uninfected males (MUI). In the meantime, based on differentially expressed miRNAs, we also predicted the target genes of these miRNAs. Revealing the *Wolbachia*-induced microRNA will help us to further understand the interactions between *Wolbachia* and its host.

## Materials and Methods

### *Laodelphax striatellu*s

The *L. striatellus* that naturally infected *Wolbachia* were collected from Nanjing, Jiangsu province of China, in 2011. After rearing in laboratory for several generations, some individuals of this population were treated with the tetracycline hydrochloride solution (0.1%) that was added to the rice seedlings for 3–4 generations until no *Wolbachia* was detected by diagnostic PCR detection. Then, both *Wolbachia*-uninfected and *Wolbachia*-infected *L. striatellus* used in this study were long-term kept in our laboratory. These lines were reared in clear plastic cups (150 mm in height and 110 mm in diameter) which were covered with gauze and contained rice seedlings. Then these planthoppers were maintained in an artificial climate chamber (temperature: 27 ± 1°C, relative humidity: 60 ± 10%, and under 16 h light: 8 h dark photoperiod). Infection status of each strain was confirmed by using PCR method to detected the *wsp* gene of *Wolbachia* with 81F (TGG TCC AAT AAG TGA TGA AGA AAC) and 691R (AAA AAT TAA ACG CTA CTC CA) primers (Braig et al., 1998).

### Small RNA Library Construction and Illumina Sequencing

In order to avoid the impact of mating, the 5-th-instar nymphs were single reared in glass test tubes (180 mm in height and 18 mm in diameter) which were covered with gauze and contained rice seedlings, and were observed every 8 h. Since the 3-day-old adults have shown sexual maturity and have the ability to mate, and the density of *Wolbachia* shows an increase in adult bodies from 0 to 4 days (Noda et al., 2001), so the 3-day-old adults were selected for RNA extraction. After they emerged, 3-day-old females (5 individuals) and males (10 individuals) adults were collected in 1.5 mL centrifuge tube, respectively. All samples were quickly frozen in liquid nitrogen, then stored at −80°C for RNA extractions. Total RNA was extracted from every sample using Trizol Reagent (Invitrogen, Carlsbad, CA, United States) according to the manufacturer’s instructions (recommended protocol). To ensure that the use of qualified samples for sequencing, RNA integrity was evaluated using the RNA Nano 6000 Assay Kit of the Agilent Bioanalyzer 2100 system (Agilent Technologies, CA, United States).

A total amount of 2.5 μg RNA per sample was used for sequencing. The sequencing libraries were created using NEB Next Ultra small RNA Sample Library Prep Kit (NEB, United States) following manufacturer’s recommendations and index codes were added to attribute sequences to each sample. The PAGE gel was used for electrophoretic fragment screening purposes, and the small RNA library obtained as the pieces was recovered by gelatinization. The clustering of the index-coded samples was operated on a cBot Cluster Generation System using TruSeq PE Cluster Kit v4-cBot-HS (Illumia), then the library preparations were sequenced on an Illumina Hiseq 2500 platform. The dataset of this study has been deposited in the Sequence Read Archive (SRA) database of NCBI with accession number PRJNA530287.

### Bioinformatics Analysis of Sequencing Data

To obtain clean reads from raw reads having low-quality, raw reads of fastq format were firstly processed through in-house perl scripts. In this step, clean reads were obtained by removing reads containing base N (N is an unrecognized base) content of 10% or more, with 5′ adapter contaminants, without 3′ adapter, and low-quality reads, and cut off the 3′ adapter sequence from raw data. And reads were trimmed and cleaned by removing the sequences smaller than 18 nt or longer than 30 nt. At the same time, Q20, Q30, GC-content and sequence duplication level of the clean data were calculated. Using Bowtie software (v1.0.0) (Langmead et al., 2009), sequence alignment of clean reads with Silva database,^1^ GtRNAdb database,^2^ Rfam database^3^ and Repbase database,^4^ filtering ribosomal RNA (rRNA), transport RNA (tRNA), nuclear small RNA (snRNA), nucleolar small RNA (snoRNA) and other ncRNAs and repeats, obtain unannotated readings containing miRNA. Sequence alignment of unannotated reads using Bowtie software to obtain positional information on the reference gene, which is a map read. In the known miRNA identification, the mapped reads were aligned with the sequence of the mature miRNA in miRBase^5^ database. In the novel miRNA identification, based on the biometric characteristics of miRNA, the miRDeep2 tool (v2.0.5) (Friedlander et al., 2012) was used to obtain possible precursor sequences. And the precursor structure energy information and miRNA secondary structure for the prediction of novel miRNAs. For the name of the novel miRNAs which first reported in *L. striatellus*, the code “lst-miRn” followed by a number assigned to the novel miRNA as number designator was used (Li et al., 2015).

### Differential Expression Analysis of Between *Wolbachia*-Uninfected and *Wolbachia*-Infected *L. striatellus*

The expression levels of miRNAs between *Wolbachia*-uninfected and *Wolbachia*-infected *L. striatellus* [FI and FUI (control), MI and MUI (control)] were analyzed using the IDEG6 in this study (Romualdi et al., 2003). The expression of miRNA in the four libraries was normalized to transcripts per million (TPM) on the basis of the following formula: Normalized expression = actual miRNA count/total count of clean reads × 10^6^. The *p* value was adjusted using *q* value (Storey, 2003). The *q* value < 0.005 and |log2 (fold change)| ≥ 1 was set as the threshold for significantly differential expression.

### Verification the Expression of *Wolbachia*-Responsive miRNAs and Target Genes Prediction via qRT-PCR

In order to further verify the expression of each miRNA, reverse transcription was carried out using the miRNA cDNA Synthesis Kit (Kang Wei Century). The miRNA was quantified according to the instructions of the miRNA qPCR Assay Kit (Kang Wei Century) following the program: 95°C for 10 min, 45 cycles of 95°C for 15 s, 64°C for 1 min, and the U6 gene was used as an internal reference gene. Specific forward primers were designed based on mature sequence (Supplementary Table S1), and the reverse primer was provided by the kit. For the target gene, specific primers were designed based on CDs sequences of related *L. striatellus* genes on line^6^ (Supplementary Table S2), reverse transcription was performed using the Prime Script^TM^ RT (TaKaRa) kit. The quantitative real time polymerase chain reaction (qRT-PCR) was using NovoStart^®^ SYBR qPCR SuperMix Plus reaction kit (Novoprotein, China) following the program: 95°C for 1 min, 39 cycles of 95°C for 20 s, 60°C for 1 min. The relative expression levels were normalized by *L. striatellus* ARF (ADP-ribosylation factor-like protein 2) gene which was a stably expressed internal reference gene, and it was recommended to be used in qPCR in *L. striatellus* (He et al., 2014). All reaction quantitative reactions were carried out in a fluorescence quantitative gradient PCR instrument qTOWER3.0 Real-Time System (Analytik Jena, Germany). Three biologic replicates were performed for each experiment. The relative expression level of each gene was calculated using the 2^–ΔΔ*Ct*^ method (Livak and Schmittgen, 2001). Relative transcriptional levels were analyzed with the SPSS v.22.0 software (SPSS, Chicago, IL, United States), independent *t* test was used to analysis the differences between groups. The *p* value of <0.05 was considered statistically significant.

### MicroRNA Target Prediction and Function Analysis

Target genes of *Wolbachia*-responsive miRNAs were predicted using our transcriptome data of *L. striatellus* by miRanda (v3.3a) (Betel et al., 2008) and RNAhybrid (v2.1.1) (Rehmsmeier et al., 2004) based on the gene sequence information of known miRNAs and newly predicted miRNAs. Target gene were annotated by aligning their sequences with NR (Deng et al., 2006), Swiss-Prot (Apweiler et al., 2004), GO (Ashburner et al., 2000), KEGG (Kanehisa et al., 2004), and Pfam (Eddy, 1998) databases using BLAST software.

## Results

### Overview Over sRNAs Sequencing

To identify *Wolbachia*-responsive miRNAs in *L. striatellus*, four sRNA libraries (FUI, FI, MUI, and MI) were constructed using high-throughput Illumina sequencing platform. In total, 17,772,698; 28,351,576; 24,916,579 and 24,311,953 raw reads were obtained, respectively. For the raw data, the reads shorter than 18 nt or longer than 30 nt were discarded. After removing low-quality sequences, 16,616,711; 26,020,481; 23,702,808 and 22,471,936 clean reads were obtained in the FUI, FI, MUI, and MI libraries, respectively, for further analysis. In all four libraries, the ratio of rRNA reads was less than 10%. The lowest rRNA read ratio appeared in the infected male sequencing library (MI, 3.69%), and overall, the proportion of rRNA read were lower in the male sequencing libraries than in the female (Supplementary Table S3). Subsequently, the proportions of common and specific sRNAs between pairs of libraries were further analyzed (Supplementary Figure S1). For the total reads, the results indicated that the number of sRNAs shared by any two libraries accounted for more than 75% of the total sRNAs, while the library-specific sRNAs accounted for only 5.56–16.55%. After removing the redundant read, the Uniq-sRNA shared between the two samples accounted for 11.45–16.14%, while the library-specific sRNA type accounted for more than 25% in all comparative groups.

### Identification and Analysis of miRNAs From Sequencing Libraries

**FIGURE 1:**
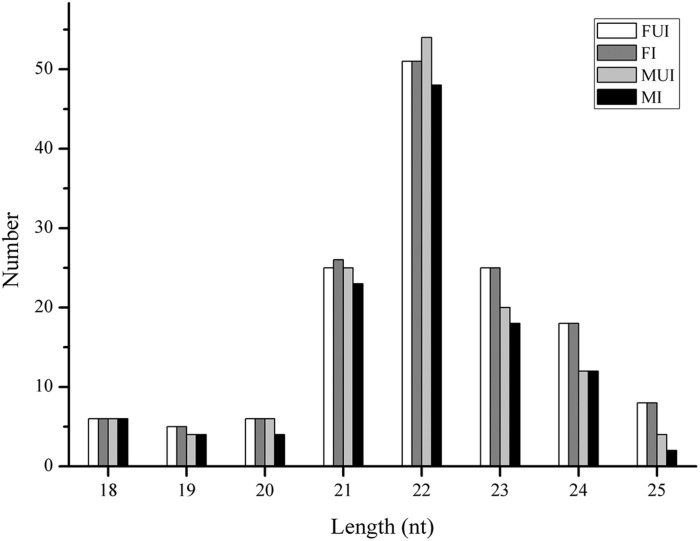
Length distribution of miRNAs in the four sequencing libraries of *L. striatellus*. FUI, female uninfected; FI, female infected; MUI, male uninfected; MI, male infected; nt, nucleotides.

To identify conserved and novel miRNAs in *L. striatellus*, the filtered small RNA sequences were analyzed. After compared with known miRNAs from miRBase database and predicted novel miRNA using miRDeep2 software, 152 miRNAs were annotated in the four sequencing libraries, which include 49 known and 103 novel miRNAs (Table 1). The length distribution of all miRNAs in the four libraries was 18–25 nt, and it was mainly distributed in 21–24 nt, with 22 nt as the largest number of miRNAs (Figure 1), which in accordance with the typical sizes of Dicer processing products (Ambros et al., 2003). The miRNA name, the mature sequences, the precursor sequence and the number of reads of each miRNA were shown in Supplementary Table S4. Familial analysis of known miRNAs and novel miRNAs of the four libraries based on sequence similarity, a total of 17 novel miRNAs and 49 known miRNAs were divided into 30 families.

**TABLE 1:** Numbers of miRNAs in the four libraries of *L. striatellus*.

**Samples**	**Known-miRNAs**	**Novel-miRNAs**	**Total**
FUI	48	96	144
FI	49	96	145
MUI	49	78	127
MI	49	68	117
Total	49	103	152

**FIGURE 2:**
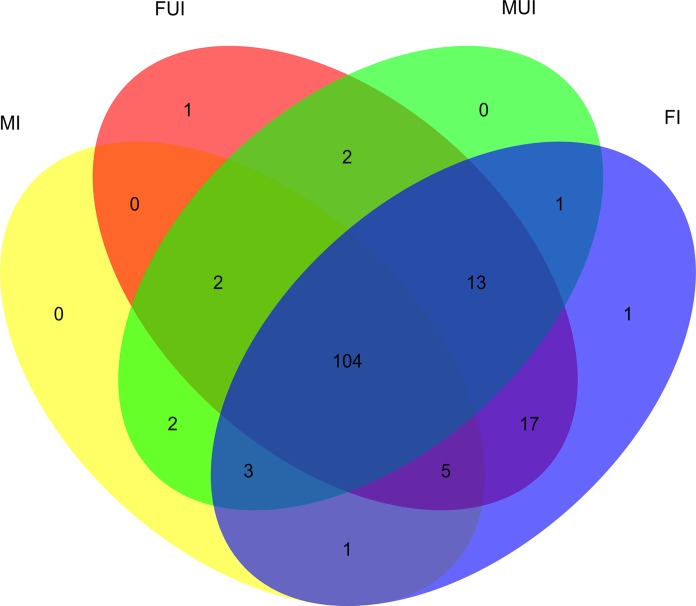
Venn diagram of unique and commonly expressed miRNAs among four sequencing libraries of *L. striatellus* (FI, FUI, MI, and MUI). Yellow oval represents MI; red oval represents FUI; green oval represents MUI; blue oval represents FI.

Totally, 104 miRNAs were shared in the four libraries (Figure 2). For females, 48 known and 91 novel miRNAs were shared, and there were 6 and 5 specifically expressed miRNAs in *Wolbachia* infected and uninfected females, respectively. Meanwhile, for males, 49 known and 62 novel miRNAs were shared, and there were 6 and 16 specifically expressed miRNAs in *Wolbachia* infected and uninfected males, respectively.

### Abundance of miRNAs

In order to clarify the highly abundant miRNAs in every library, top twenty most abundant miRNAs expressed in each of libraries were shown in Table 2 (accounting for more than 90% of total miRNA reads), 16 of which expressed in the four libraries were shared, including two newly identified miRNAs (lst-miR-n74-5p and lst-miR-n97-5p). The two most abundantly expressed miRNAs of the four sequenced libraries were lst-miR-1-3p and lst-miR-184-3p.

**TABLE 2:** Top 20 most abundant miRNAs expressed in the four sequencing libraries of *L. striatellus* (TPM data were shown).

**miRNA**	**FI**	**miRNA**	**FUI**	**miRNA**	**MI**	**miRNA**	**MUI**
lst-miR-1-3p	311,986	lst-miR-1-3p	305,001	lst-miR-1-3p	252,453	lst-miR-1-3p	379,158
lst-miR-184-3p	131,686	lst-miR-184-3p	98,290	lst-miR-184-3p	88,093	lst-miR-184-3p	103,236
**lst-miR-n74-5p**	**69,022**	lst-miR-7-3-5p	66,377	lst-miR-7-3-5p	82,894	lst-miR-7-3-5p	66,256
lst-miR-7-3-5p	48,993	**lst-miR-n74-5p**	**62,449**	lst-miR-7-1-5p	80,591	lst-miR-7-1-5p	65,586
lst-miR-7-1-5p	44,779	lst-miR-7-1-5p	62,145	lst-miR-7-2-5p	80,587	lst-miR-7-2-5p	65,586
lst-miR-7-2-5p	44,779	lst-miR-7-2-5p	62,145	lst-miR-7-4-5p	80,587	lst-miR-7-4-5p	65,586
lst-miR-7-4-5p	44,779	lst-miR-7-4-5p	62,145	**lst-miR-n74-5p**	**45,660**	**lst-miR-n97-5p**	**42,633**
**lst-miR-n97-5p**	**30,647**	**lst-miR-n97-5p**	**35,988**	**lst-miR-n97-5p**	**31,039**	**lst-miR-n74-5p**	**25,789**
lst-miR-281-4-3p	23,360	lst-miR-281-4-3p	20,358	lst-miR-10-1-5p	23,499	lst-miR-10-1-5p	15,606
lst-miR-281-3-3p	23,357	lst-miR-281-3-3p	20,354	lst-miR-316-1-5p	17,410	lst-miR-281-3-3p	12,390
lst-miR-281-1-3p	23,311	lst-miR-281-1-3p	20,326	lst-miR-10a-3p	15,294	lst-miR-281-4-3p	12,390
lst-miR-750-2-3p	14,211	lst-miR-10-1-5p	13,487	lst-miR-10a-2-5p	15,294	lst-miR-281-1-3p	12,354
lst-miR-281-2-3p	13,472	lst-miR-281-2-3p	11,288	lst-miR-10a-3-5p	15,294	lst-miR-10a-3p	9847
lst-miR-10-1-5p	13,320	lst-miR-750-2-3p	10,839	lst-miR-281-4-3p	14,186	lst-miR-10a-2-5p	9847
lst-miR-275-1-3p	13,285	lst-miR-275-1-3p	10,252	lst-miR-281-3-3p	14,181	lst-miR-10a-3-5p	9847
lst-miR-275-2-3p	13,285	lst-miR-275-2-3p	10,252	lst-miR-281-1-3p	14,087	lst-miR-750-2-3p	8827
lst-miR-750-1-3p	13,267	lst-miR-750-1-3p	9909	lst-miR-750-2-3p	12,996	lst-miR-316-1-5p	8814
lst-miR-316-1-5p	9759	lst-miR-316-1-5p	9409	lst-miR-750-1-3p	11,672	lst-miR-750-1-3p	8143
lst-miR-279-3p	8745	lst-miR-10a-3p	8530	lst-miR-10-2-5p	9246	lst-miR-275-1-3p	6708
lst-miR-278-3p	8570	lst-miR-10a-2-5p	8530	lst-miR-275-1-3p	8012	lst-miR-275-2-3p	6708

*Novel miRNAs found in this study were shown in bold.*

### Differentially Expressed miRNA of *L. striatellus* in Response to *Wolbachia* Infection

**FIGURE 3:**
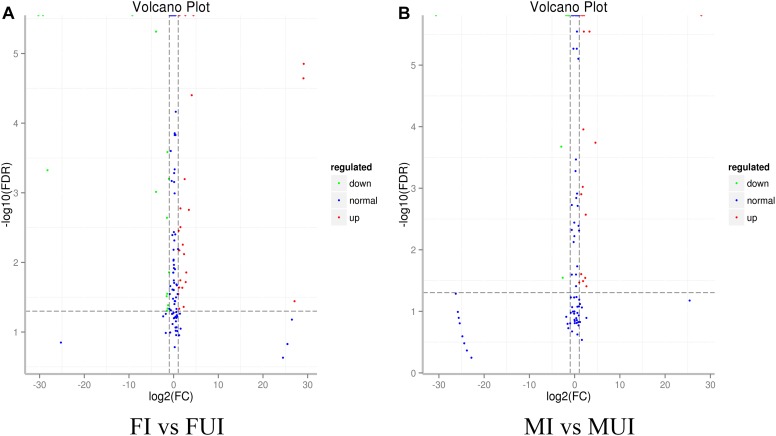
Volcano maps of differential miRNAs analysis between *Wolbachia*-infected and *Wolbachia*-uninfected *L. striatellus* (**A**: Comparison between females; **B**: Comparison between males). Each point represents a miRNA, red dots indicate significantly up-regulated miRNAs, and green dots indicate significantly down-regulated miRNAs, blue dots indicate no significant difference in miRNAs.

**FIGURE 4:**
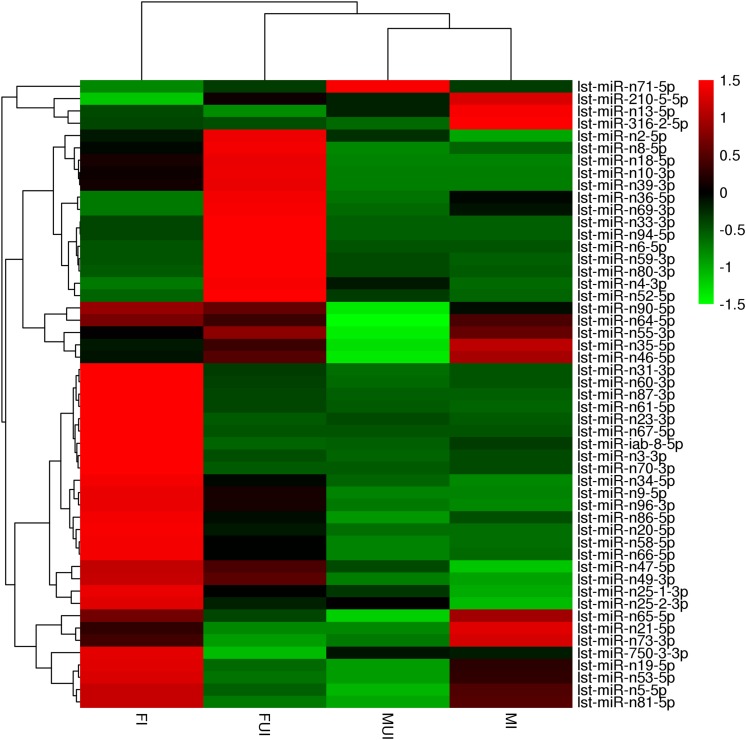
Heat map showing the differentially expressed miRNAs in four libraries (FUI, FI, MUI and MI) of *L. striatellus*. The fold-change ratios of the miRNAs indicated by the different colors, red indicates higher levels of miRNAs and green indicates lower levels of miRNAs.

**FIGURE 5:**
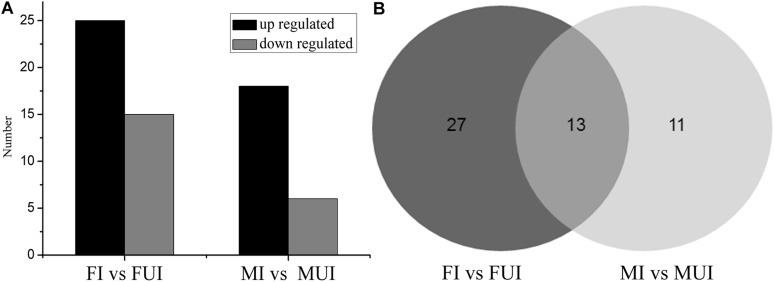
The statistics of differentially expressed miRNAs in two comparisons of *L. striatellus* (FI vs. FUI and MI vs. MUI). **(A)** Contrast between up-regulated and down-regulated differentially expressed miRNAs in two comparisons of *L. striatellus*. **(B)** Venn diagram of differentially expressed miRNAs that were shared and unique in two comparisons of *L. striatellus*.

To reveal differentially expressed miRNAs in response to *Wolbachia*-infection, the expression levels of these miRNA were compared in infected and uninfected females and males of *L*. *striatellus*, respectively. A volcano plot of the different expressed miRNAs was shown in Figure 3. Overall, compared with uninfected individuals, there were 51 differently expressed miRNAs including 4 known and 47 novel miRNAs in *Wolbachia*-infected females and males (Figure 4). Compared with the uninfected females, 25 miRNAs were up-regulated and 15 miRNAs were down-regulated in *Wolbachia* infected females. By contrast, 18 miRNAs were up-regulated and 6 miRNAs were down-regulated in MI compared with the MUI (Figure 5A). Apparently, *Wolbachia* induced more differentially expressed miRNAs in females than males (about 1.67-fold), suggesting that *Wolbachia* infection may have a broader effect on females (Figure 5). In *T. urticae*, *Wolbachia* infection also induced more differentially expressed miRNAs in females (Rong et al., 2014).

Interestingly, 13 differentially expressed miRNAs were shared in the comparisons of female and male (Figure 5B and Table 3). Among them, three miRNAs were down-regulated and 8 miRNAs were up-regulated in both females and males after infection with *Wolbachia*. The miRNA lst-miR-n21-5p showed the highest degree of up-regulation in *Wolbachia*-infected females and males, and both lst-miR-n4-3p and lst-miR-n52-5p showed the highest degree of down-regulation in *Wolbachia*-infected females and males. Besides, lst-miR-n25-2-3p showed up-regulation in females, but was down-regulated in males. On the contrary, lst-miR-n8-5p showed down-regulation in females, but was up-regulated in males. These data show that the miRNAs in response to *Wolbachia*-infected in both sexes of *L. striatellus* which might have connections and differences.

**TABLE 3:** Common *Wolbachia*-responsive miRNAs in the female and male comparisons of *L. striatellus*.

**miRNA name**	**FI^a^**	**FUI^a^**	**log_2_ (FI/FUI)**	**MI^a^**	**MUI^a^**	**log_2_ (MI/MUI)**
lst-miR-n86-5p	76.9559043	31.5197649	1.287774	20.19771	7.379611	1.452576
lst-miR-n31-3p	219.207727	35.4597355	2.628046	20.19771	3.689805	2.452576
lst-miR-n19-5p	65.2959188	11.8199118	2.465775	35.90705	3.689805	3.282651
lst-miR-n73-3p	30.3159623	7.87994122	1.94383	44.88381	11.80738	1.926504
lst-miR-n5-5p	1070.38667	429.456796	1.317547	807.9085	247.217	1.708414
lst-miR-n81-5p	25.6519681	3.93997061	2.702804	17.95352	0.737961	4.6045
lst-miR-n21-5p	13.9919826	0	27.06003	26.93028	0	28.00466
lst-miR-n53-5p	18.6559768	3.93997061	2.243372	11.22095	2.213883	2.341539
lst-miR-n25-2-3p	27.9839652	11.8199118	1.24338	2.24419	14.02126	−2.6433
lst-miR-n8-5p	27.9839652	78.7994122	−1.4936	8.976761	1.475922	2.60461
lst-miR-n71-5p	9.3279884	27.5797943	−1.56397	26.93028	100.3627	−1.89792
lst-miR-n4-3p	0	130.01903	−30.2761	4.488381	34.68417	−2.95001
lst-miR-n52-5p	2.3319971	1402.62954	−30.64	0	167.5172	−9.23

*^*a*^The numbers show the transcripts per million (TPM) of miRNAs.*

### Target Gene Prediction, and GO and KEGG Analyses of Differentially Expressed miRNAs

To better understand the function of *Wolbachia*-responsive miRNAs, 926 and 799 predicted target genes were predicted for the 44 differentially expressed miRNAs in female and male comparisons, respectively (Supplementary Tables S5, S6).

**FIGURE 6:**
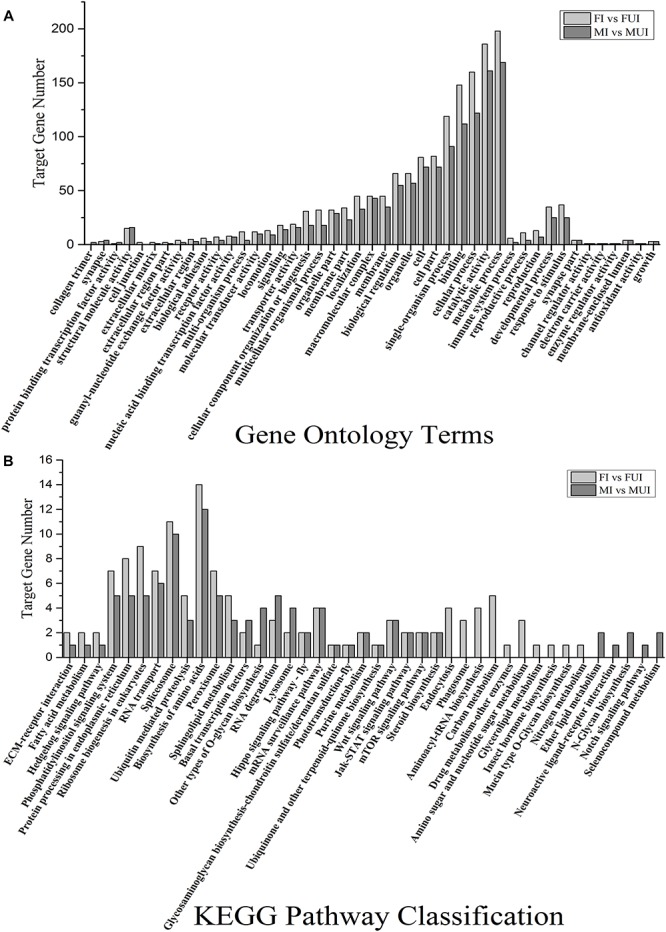
Gene ontology (GO) annotation and Kyoto Encyclopedia of Genes and Genomes (KEGG) classification of the target genes of the two comparisons in *L. striatellus*. The *y*-axis showed the number of genes, and the *x*-axis showed the GO category **(A)** and the KEGG classification **(B)**, respectively.

The GO enrichment analysis indicated that the predicted target genes related to metabolic process (198 genes in FI vs. FUI and 169 genes in MI vs. MUI), catalytic activity (186 genes in FI vs. FUI and 161 genes in MI vs. MUI), cellular process (160 genes in FI vs. FUI and 122 genes in MI vs. MUI), binding (148 genes in FI vs. FUI and 112 genes in MI vs. MUI), single-organism process (119 genes in FI vs. FUI and 91 genes in MI vs. MUI) were the most enriched categories in both comparisons (Figure 6A). Notably, most of categories in GO enrichment showed that the predicted target gene number in the female comparison were more than in the male comparison. As mentioned above, these results also suggested that *Wolbachia* infection had a broader impact on females than males. In view of previous research showed the reproduction and immunity of host might be affected by *Wolbachia*, we also found predicted genes related to immune system process, reproductive process, reproduction, developmental process, response to stimulus, antioxidant activity and growth were enriched in female and male comparisons (Figure 6A).

KEGG pathways analysis showed that the predicted target genes were annotated to 36 and 31 KEGG pathways in female and male comparisons, respectively. Among them, 26 KEGG pathways were shared in both female and male comparisons (Figure 6B), and the predicted target genes related to biosynthesis of amino acids and spliceosome were the most enriched pathways in both comparisons. In this study, we observed that target genes related to reproduction (mTOR signaling pathway), immune (lysosome, spliceosome, peroxisome, and Jak-STAT signaling pathway), and sphingolipid metabolism and steroid biosynthesis were enriched in both female and male comparisons. Intriguingly, some pathways such as endocytosis, phagosome, aminoacyl-tRNA biosynthesis, carbon metabolism, amino sugar and nucleotide sugar metabolism, glycerolipid metabolism and insect hormone biosynthesis were only enriched in the female comparison. Meanwhile, other pathways such as ether lipid metabolism, neuroactive ligand-receptor interaction, N-glycan biosynthesis, notch signaling pathway and selenocompound metabolism were only enriched in the male comparison.

### qRT-PCR Validations of Differently Expressed miRNA and Their Predicted Target Genes

**FIGURE 7:**
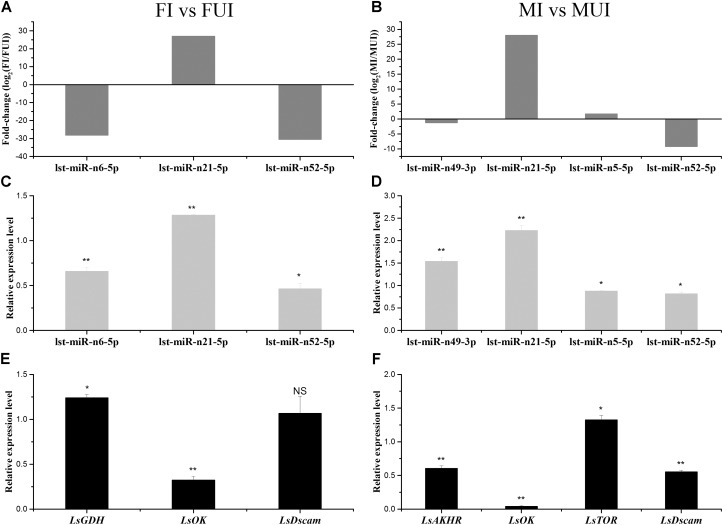
Expression patterns of five differentially expressed miRNAs and their predicted target genes in response to *Wolbachia* infection of *L. striatellus*. For the comparisons FI vs. FUI and MI vs. MUI, the relative expression levels of differentially expressed miRNAs were identified by Illumina sequencing **(A,B)** and qRT-PCR methods **(C,D)**, respectively. **(E,F)** The expression profiles of corresponding target gene of differentially expressed miRNAs were verified using qRT-PCR methods. The corresponding mRNA under each miRNA is its predicted target gene. GDH, glutamate dehydrogenase; OK, orcokinin; Dscam, Down syndrome cell adhesion molecule-like protein; AKHR, adipokinetic hormone receptor; TOR, target of rapamycin. The error bars indicate standard errors of averages from three biological replicates (NS: not significant; ^*^P < 0.05; ^∗∗^*P* < 0.01).

The quantitative real time polymerase chain reaction was conducted to further validate the expression pattern of differentially expressed miRNAs. The expression patterns of several miRNAs by Illumina sequencing and qRT-PCR were shown in Figures 7A–D. We found that the miRNA lst-miR-n52-5p was significantly down-regulated while another miRNA lst-miR-n21-5p was significantly up-regulated in both *Wolbachia*-infected females and males. In addition, the miRNA lst-miR-n6-5p was also significantly down-regulated in *Wolbachia* infected females. Although high-throughput sequencing of small RNAs could reveal a lot of useful information, it could also produce some inaccurate results. For instance, in this study, the Illumina sequencing results of these two miRNAs lst-miR-n49-3p and lst-miR-n5-5p in males were inconsistent with their qRT-PCR results. However, the expression patterns of their predicted target genes were opposite to the expression of their corresponding miRNAs (including lst-miR-n49-3p and lst-miR-n5-5p) except *LsDscam* (Figures 7E,F). We preliminarily speculate that *LsDscam* may not be the real target gene of the miRNA lst-miR-n52-5p. In summary, our RNA sequencing results could reflect the effects of *Wolbachia* infection on the expression of miRNAs and their corresponding target genes in the *L*. *striatellus*.

## Discussion

At present very little is known about the effects of *Wolbachia*-infection on host miRNA expression. In the present study, we constructed and sequenced four sRNA libraries from both sexes of *Wolbachia*-infected and *Wolbachia*-uninfected *L*. *striatellus*. The results showed that *Wolbachia* infection caused a change in the expression of miRNA in the *L. striatellus*. These differentially expressed miRNAs may be involved in multiple aspects of the biological characteristics of the host. For instance, miR-210 play a role in mitotic progression and modulating circadian outputs (He et al., 2013; Cusumano et al., 2018). We also found the miRNA lst-miR-210-5p was up-regulated in *Wolbachia*-infected male *L*. *striatellus*, which targets a ubiquitin-protein ligase E3A gene (Figure 4 and Supplementary Table S5). The ubiquitin-protein ligase E3A plays an essential role in the regulation of the circadian system in mammals and flies (Gossan et al., 2014). Although the role of these differentially expressed miRNAs were based solely on their predicted target genes, and even many of them were newly discovered, we still discussed several important differentially expressed miRNAs that supposedly affected multiple biologic aspects of its host below.

### miRNAs in Response to *Wolbachia* May Be Involved in Ecdysteroidogenesis of *L. striatellu*s

Existing research result indicated that *Wolbachia* might be involved in ecsysteroidogenesis (Negri, 2011). For instance, in filarial worms, *Wolbachia* might play a critical role in host embryogenesis and molting (Casiraghi et al., 2002; Arumugam et al., 2008). In *Eurema hecabe*, removing *Wolbachia* resulting phenotypic defects were similar to knock-out of ecdysone receptor (*EcR*) gene in *Blattella germanica* and *Drosophila melanogaster* (Negri, 2011). Here we showed that the miRNA lst-miR-n21-5p was up-regulated in *Wolbachia*-infected females and males of *L. striatellus* which target a *orcokinin* (*OK*) gene (Figures 7C,D). The *OK* gene encoded a kind of neuropeptide that has been identified in a variety of arthropods, and this gene was down-regulated in both *Wolbachia*-infected females and males, with especially lower expression level in *Wolbachia*-infected males (Figures 7E,F). In *Rhodnius prolixus*, 20-hydroxyecdysone (20E) could regulate the expression level of *OK* gene (Wulff et al., 2018), and the *OK* gene was also involved in the neuronal regulation of ecdysteroidogenesis in *Bombyx mori* (Yamanaka et al., 2011). We hypothesized that *Wolbachia* interferes with the pathway involved in ecdysteroidogenesis by regulating expression of lst-miR-n21-5p in *L. striatellus*.

In addition, our results also showed that lst-miR-n3-3p whose predicted target gene, the Halloween gene *Shade* (*Shd*, *cyp314a1*) was notably up-regulated in *Wolbachia*-infected females of *L. striatellus*. The *Shd* gene was a cytochrome P450 monooxygenase (CYP) which catalyzes the conversion of ecdysone into active 20E. In *L. striatellus*, knock-down of the *Shd* gene could decrease expression level of *EcR* gene, and also significantly decrease the titer of 20E (Jia et al., 2013; Zhai et al., 2017). Similarly, nuclear hormone receptor E75 gene, a crucial 20E response gene that affects ecdysteroid titer was also downregulated in *Wolbachia*-infected females and males of *T. urticae* (Rong et al., 2014). Taken together, our data seem to support previous hypothesis that there might be a link between *Wolbachia* and ecdysteroid signaling (Negri, 2011).

### *Wolbachia*-Responsive miRNA May Involve in Immune Response of *L. striatellu*s

It was theorized that *Wolbachia* can activate the immune system of host insect. We found that miRNA lst-miR-n52-5p was down-regulated in both *Wolbachia*-infected female and male of *L. striatellus*. Although the results of qRT-PCR indicated that *LsDscam* might not be the real target gene of this miRNA, we also found another predicted target gene, hexamerin gene which was one gene of the haemocyanin protein family (Figure 4 and Supplementary Tables S5, S6). In adults of *Riptortus pedestris*, that was infected with gut symbiont *Burkholderia*, the hexamerin-a and hexamerin-b proteins were highly expressed compared to uninfected individuals (Lee et al., 2017). The hexamerin gene was up-regulated in *Spiroplasma citri*-infected *Circulifer haematoceps*, and RNAi knockdown of hexamerin gene resulted in significant reduction in phenoloxidase-like activity, as well as increased mortality of *S. citri*-infected leafhoppers (Eliautout et al., 2016). In *Ae. aegypti*, the transcripts of three prophenoloxidase genes were up-regulated by *Wolbachia* infection (Kambris et al., 2009), phenoloxidase activity was also found significantly elevated in *Wolbachia*-infected females of *D*. *melanogaster* (Thomas et al., 2011). Therefore, it could be suspected that *Wolbachia* might enhance the expression level of hexamerin gene, and consequently, activity the phenoloxidase of *L. striatellus* through down-regulating the expression of lst-miR-n52-5p.

### Female Fecundity May Be Regulated by miRNAs That Response to *Wolbachi*a

Previous studies have shown that *Wolbachia* could enhance the fecundity of female hosts (Mazzetto et al., 2015; Rahimi-Kaldeh et al., 2017; Guo et al., 2018). In this study, we found that several target genes of differently expressed miRNAs responsing to *Wolbachia* infection were associated with female fecundity. The miRNA lst-miR-n36-5p was notably down-regulated in *Wolbachia*-infected females which targets a gene coding for vitellogenin-6 (*vg6*) (Figure 4 and Supplementary Table S5). In insect, the *vg* gene was highly expressed in the female fat body, and it could significantly affect on oviposition and egg hatchability (Ali et al., 2017; Zhang et al., 2017). Interestingly, lst-miR-n10-3p which targets nuclear hormone receptor Fushi tarazu-factor 1 beta (β*FTZ-F1*) gene was also observed down-regulated in *Wolbachia*-infected females (Figure 4 and Supplementary Table S5). In *Drosophila*, knockdown of β*FTZ-F1* gene could prevent juvenile hormone (JH) activation, whereas overexpression enhanced the activation of JH (Dubrovsky et al., 2011). We speculate that *Wolbachia* appears to enhance the fecundity of *L. striatellus* by down-regulating the expression of lst-miR-n36-5p and lst-miR-n10-3p in female hosts.

In *L. striatellus*, it has already confirmed that *Wolbachia* infection can increase the fecundity of females (Guo et al., 2018). The results reported in this study might help to further reveal the cause of this phenomenon.

### Male Fertility May Be Regulated by miRNAs in Response to *Wolbachi*a

Although cytoplasmic incompatibility is the most famous reproductive phenotype caused by *Wolbachia* in arthropod, the mechanism of this phenomenon is still unclear. However, more and more researches about *Wolbachia* infection affecting on fertility of male host have been reported (Liu et al., 2014; Ju et al., 2017). In this study, we also annotated several differently expressed miRNAs whose predicted target gene were concerned with male fertility. For instance, the miRNA lst-miR-n5-5p that targets a gene coding target of rapamycin (*TOR*) was observed down-regulated in *Wolbachia*-infected males (Figures 7D,F). A growing number of studies show that the mammalian target of rapamycin (mTOR) signaling pathway plays a crucial role in spermatogenesis (Schell et al., 2016; Serra et al., 2017). Another fertility-related miRNA gene, lst-miR-n13-5p was also observed up-regulated in *Wolbachia*-infected males, and it targets a gene coding juvenile hormone esterase (JHE) (Figure 4 and Supplementary Table S6). JHE play an important role in the regulation of JH titer (Mackert et al., 2008; Tsubota et al., 2010). In *D. melanogaster*, the researchers also found that over-expression of the Juvenile hormone-inducible protein 26 (*JhI-26*) gene in *Wolbachia*-uninfected males resulted in a significant reduction in egg hatching rates after mating with *Wolbachia*-uninfected females, and that *Wolbachia*-infected females could rescue egg hatching (Liu et al., 2014). This result show that the occurrence of fertility might related to the changes of juvenile hormone levels. It is possible that *Wolbachia* down-regulates the expression of *jhe* gene to interfere the JH pathway by up-regulate the lst-miR-n13-5p in males, thereby possibly induce paternal defects in fertility. In addition, the miRNA lst-miR-n81-5p was also up-regulated in *Wolbachia*-infected males, it targets a sperm-associated antigen 6 (*Spag6*) gene (Figure 4 and Supplementary Table S6). The *Spag6* initially found in human testis and it was essential for sperm motility and male fertility (Sapiro et al., 2002). The down-regulation of lst-miR-n81-5p maybe in associated with decreased fertility.

### Redox Homeostasis of *L. striatellus* May Be Regulated by miRNAs in Response to *Wolbachi*a

It was considered that *Wolbachia* regulated redox homeostasis to maintain their relationship with host (Zug and Hammerstein, 2015). The miRNA lst-miR-n23-3p was likely to involve in oxidation-reduction reactions and showed up-regulated in *Wolbachia*-infected females which targets a gene coded mitochondrial manganese superoxide dismutase (*mMnSOD*) (Figure 4 and Supplementary Table S5). Similarly, *Wolbachia* infection significantly reduced SOD activity in the larvae of *D. melanogaster* (Wang et al., 2012). The miRNA lst-miR-n47-5p was found down-regulated in *Wolbachia*-infected males that targets a gene coded thioredoxin reductase (*TrxR*) (Figure 4 and Supplementary Table S6). The expression of *TrxR* gene was up-regulated in varying degrees after *M*. *anisopliae* and *E. coli* infection of *Helicoverpa armigera* (Zhang et al., 2015). Interestingly, silencing *TrxR* gene caused a significant decrease in the native bacterial load of the ticks in both the midgut and salivary glands (Budachetri and Karim, 2015). In *L. striatellus*, native *Wolbachia* may utilize *mMnSOD* and *TrxR* to better coexist with its host.

## Conclusion

In summary, this study provides the first information on miRNAs of *L. striatellus* responding to *Wolbachia*. Our results suggest that *Wolbachia* may manipulate the physiological processes of *L. striatellus* by using miRNAs of host, and these results could contribute to further insight into the mechanisms of *Wolbachia*-host interaction. However, there were no confirmed targets for *L. striatellus* miRNAs in our research, future research is necessary to confirm interaction between these miRNAs and their predicted gene targets.

## Data Availability

Publicly available datasets were analyzed in this study. This data can be found at https://www.ncbi.nlm.nih.gov/sra.

## Ethics Statement

The research project was conducted on insect pest species that are not subjected to any specific ethical issue and legislation.

## Author Contributions

K-JZ, LL, and HL conceived and designed the study. LL and K-JZ performed the experiments, analyzed the data, and drafted the manuscript. XR, Y-YL, and HL participated in manuscript drafted and modification. All authors read and approved the final manuscript.

## Conflict of Interest Statement

The authors declare that the research was conducted in the absence of any commercial or financial relationships that could be construed as a potential conflict of interest.

## Footnotes

1 http://www.arb-silva.de/

2 http://lowelab.ucsc.edu/GtRNAdb/

3 http://rfam.xfam.org/

4 http://www.girinst.org/repbase/

5 http://www.mirbase.org/

6 http://bioinfo.ut.ee/primer3/

## Supplementary Material

FIGURE S1 Venn diagram illustrating the numbers and percentages of total and unique reads between various comparisons of *L. striatellus*. Total sRNA, total number of sRNA reads; Unique sRNA, numbers of sRNA types.

TABLE S1 The primers used for reverse transcription quantitative PCR (RT-qPCR) of miRNA.

TABLE S2 The primers used for reverse transcription quantitative PCR (RT-qPCR) of predicted target genes.

TABLE S3 Statistics of sequencing data in the four libraries of *L. striatellus*.

TABLE S4 Description of the 49 conserved and 103 newly miRNAs identified in *L. striatellus*.

TABLE S5 Predicted target genes that may be reverse regulated by differentially expressed miRNAs in the female comparison.

TABLE S6 Predicted target genes that may be reverse regulated by differentially expressed miRNAs in the male comparison.
