# Supplementary figures and images for: Identification of Wolbachia-Responsive miRNAs in the Small Brown Planthopper, Laodelphax striatellus
Source: Front Physiol. 2019 Jul 24;10:928. doi: 10.3389/fphys.2019.00928 (PMC6668040; doi:10.3389/fphys.2019.00928)

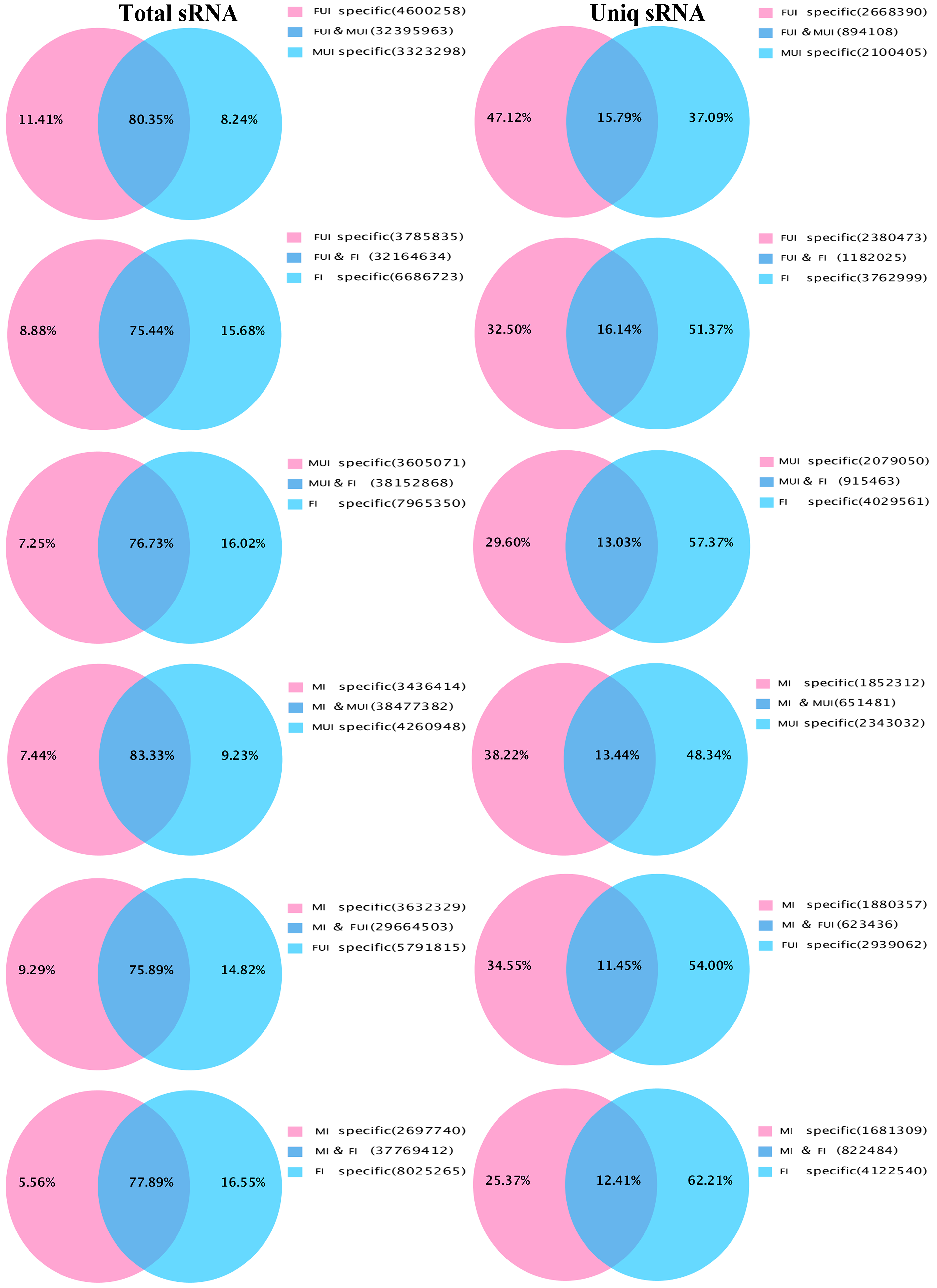

Supplement: FIGURE S1 — Venn diagram illustrating the numbers and percentages of total and unique reads between various comparisons of L. striatellus. Total sRNA, total number of sRNA reads; Unique sRNA, numbers of sRNA types. [file Image_1.TIF]
