# Supplementary material for: Identification of Wolbachia-Responsive miRNAs in the Small Brown Planthopper, Laodelphax striatellus
Source: Front Physiol. 2019 Jul 24;10:928. doi: 10.3389/fphys.2019.00928 (PMC6668040; doi:10.3389/fphys.2019.00928)
Supplement: TABLE S1 — The primers used for reverse transcription quantitative PCR (RT-qPCR) of miRNA. [file Table_1.docx]

**Table S1.** The primers used for reverse transcription quantitative PCR (RT-qPCR) of miRNA.

| miRNA | Primer sequence (5’–3’) |
| --- | --- |
| LsU6F | GCGCAAGGATGACACGCAAA |
| lst-miR-n5-5p | GGTACAGTCTGGTGTTGTACAGGAGG |
| lst-miR-n52-5p | GGTGCCTTTGGGTCTGTCCA |
| lst-miR-n21-5p | GGCCCATGTTGTGTGGATTTTTTTTGGC |
| lst-miR-n49-3p | CGGCCCGGAGGACCAA |
| lst-miR-n6-5p | GGCACTGCTGTATATTGACGGGGA |
