# Supplementary material for: Identification of Wolbachia-Responsive miRNAs in the Small Brown Planthopper, Laodelphax striatellus
Source: Front Physiol. 2019 Jul 24;10:928. doi: 10.3389/fphys.2019.00928 (PMC6668040; doi:10.3389/fphys.2019.00928)
Supplement: TABLE S2 — The primers used for reverse transcription quantitative PCR (RT-qPCR) of predicted target genes. [file Table_2.docx]

**Table S2.** The primers used for reverse transcription quantitative PCR (RT-qPCR) of predicted target genes.

| Gene name | Direction | Primer sequence (5’–3’) |
| --- | --- | --- |
| *LsDscam* | Forward | AACGTCATCTGCCACCTACA |
| *LsDscam* | Reverse | CCCACTGACAGACACTCCAT |
| *LsAKHR* | Forward | TCTTGGCGAGATCTACCGTC |
| *LsAKHR* | Reverse | GAGTTGGTGCACGCGAATAT |
| *LsTOR* | Forward | CATCAGCAGTCGTCCATTGG |
| *LsTOR* | Reverse | CCAAAGTGTCAACAGGCGAA |
| *LsOK* | Forward | CGCTGGTAGTCTGTCTTGGA |
| *LsOK* | Reverse | TGTCCCAACACCATTCCTGA |
| *LsGDH* | Forward | AAGGATTCATTGGACCCGGT |
| *LsGDH* | Reverse | CCCTGGTTGATTGGCTTTCC |
| *LsARF* | Forward | TTGGACAGTATCAAGACCCATC |
| *LsARF* | Reverse | GCAGCAATGTCATCAATAAGC |
