# Supplementary material for: Identification of Wolbachia-Responsive miRNAs in the Small Brown Planthopper, Laodelphax striatellus
Source: Front Physiol. 2019 Jul 24;10:928. doi: 10.3389/fphys.2019.00928 (PMC6668040; doi:10.3389/fphys.2019.00928)
Supplement: TABLE S3 — Statistics of sequencing data in the four libraries of L. striatellus. [file Table_3.docx]

**Table S3. Statistics of sequencing data in the four libraries of *L. striatellus*.**

| Samples | FUI | FI | MUI | MI | Total |
| --- | --- | --- | --- | --- | --- |
| Raw_reads | 17772698 | 28351576 | 24916579 | 24311953 | 95352806 |
| Clean_reads | 16616711 | 26020481 | 23702808 | 22471936 | 88811936 |
| rRNA | 1593262 | 2578222 | 1395941 | 829583 | 6397008 |
